# Laser Ablation Synthesis in Solution and Nebulization of Silver-109 Nanoparticles for Mass Spectrometry and Mass Spectrometry Imaging

**DOI:** 10.1021/acsmeasuresciau.1c00020

**Published:** 2021-08-25

**Authors:** Aneta Płaza, Artur Kołodziej, Joanna Nizioł, Tomasz Ruman

**Affiliations:** † Doctoral School of Engineering and Technical Sciences at the Rzeszów University of Technology, 8 Powstańców Warszawy Ave., Rzeszów 35-959, Poland; § Rzeszów University of Technology, Faculty of Chemistry, Inorganic and Analytical Chemistry Department, 6 Powstańców Warszawy Ave., 35-959 Rzeszów, Poland

**Keywords:** silver-109 monoisotopic nanoparticles, laser ablation synthesis in solution, low molecular weight compounds, mass spectrometry, matrix-free laser desorption/ionization, surface-assisted desorption/ionization

## Abstract

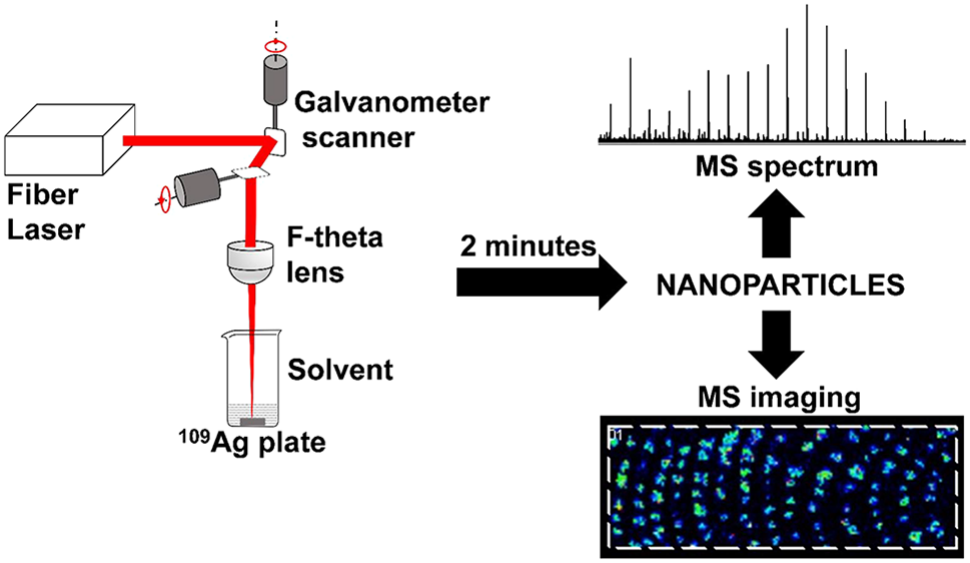

Preparation of monoisotopic silver-109 nanoparticles (^109^AgNPs) by pulsed fiber laser (PFL) ablation synthesis in solution (LASiS) with the use of a 2D galvoscanner (2D GS) is described. The procedure of covering of custom-made stainless-steel MALDI targets containing studied objects via nebulization is also presented. Examples of application of the new method (PFL-2D GS LASiS and nebulization) in mass spectrometry (MS) analyses and MS imaging (MSI) are shown. These include tests with a nonionic nucleoside and saccharide, ionic amino acids, and also a low-molecular-weight polymer. Fingerprint MS imaging is shown as an example of a fast and simple MSI procedure.

## Introduction

One of the most utilized laser mass spectrometry methods is matrix-assisted laser desorption/ionization mass spectrometry (MALDI MS), which was developed by Tanaka et al. in 1988.^1^ It offers soft ionization potential, being therefore a powerful analytical tool for the analysis of ionic high-molecular-weight molecules, such as peptides, proteins, and DNA/RNA,^2−4^ but also, it is useful for detection of some nonionic classes of chemical compounds such as lipids, etc.^5−7^ However, MALDI has not been too often applied to detect low-molecular-weight (LMW) compounds (MW < 1000 Da), because MALDI matrices are low-molecular-weight organic acids and produce a variety of matrix-related ions during the desorption/ionization process, which complicates the spectrum and causes suppression of analyte peaks.^8−10^

Discussed problems have been partly solved by the development of surface-assisted desorption/ionization (SALDI) mass spectrometry techniques, where target plates are coated with various nanostructures.^7,11−17^ Applications of nanoparticles not only allow reduction of spectral interference but also simplify the mass spectrum. The sample preparation step is also much simpler; usually only application of sample is required.^16,17^ What is more, methods based on nanostructures produce very good spot-to-spot reproducibility and greatly reduce the “sweet-spot” problem.^13^

The literature describes two main approaches for the synthesis of nanostructures: the top-down approach, where a larger structure is broken down into NPs, and the bottom-up approach, in which material is synthesized from the molecular or atomic level.^18,19^ Chemical reduction is classified as a bottom-up approach and is one of the most common strategies in use for the synthesis of nanoparticles for experiment MS.^17,20,21^ However, chemical purity problems arise due to the use of substances for chemical reactions such as metal precursors, reducing agents, stabilizers, and oxidized products, which are the source of reagent-related ions and yield numerous interfering signals.^22−25^

The above-mentioned problem was solved with the application of laser ablation synthesis in solution (LASiS) for the production of nanoparticles.^26^ LASiS employs pulsed laser irradiation to ablate a solid material target submerged in liquid, ejecting NPs from the plasma plume into the surrounding solution.^27^ This method allows for stabilizer- and reducing-agent-free NP production.^26−29^ LASiS produces nanoparticle suspensions of a relatively high chemical purity compared to chemical methods.^30^

This study describes a new method of production of chemically pure silver-109 nanoparticles in suspension with an application method for covering of studied objects or surfaces. For the first time, 1064 nm pulsed fiber laser (PFL) with 2D galvanometer scanner (2D GS) is shown as a very good source of nanoparticles. The GS module allows very precise and fast scanning of a focused laser beam on the sample surface, virtually removing any heat buildup or local melting of the ablation target. It allows very efficient and fast production of relatively big amounts of nanoparticles compared to systems without it. Moreover, the GS module allowed us to use a 20 W pulsed fiber laser directly on the ablation target with a full laser frequency of 80 kHz. Generated silver-109 nanoparticles are shown to be highly useful for LDI mass spectrometry and also mass spectrometry imaging (MSI). This work presents LDI MS results for test compounds belonging to groups such as amino acids, saccharides, nucleosides, and polymers as well as MSI results for the fingerprint.

## Experimental Section

### Materials

A silver-109 isotope of 99.7% isotopic purity was bought from Trace Sciences International (USA). l-Histidine and d-ribose were purchased from Sigma-Aldrich (99% purity). Thymidine was purchased from Alfa Aesar (99% purity). Poly(propylene glycol) (PPG, average Mn 1000 Da) was purchased from Sigma-Aldrich. All solvents were of HPLC quality, except for water (18 MΩ cm water produced locally). Steel targets were machined from H17 stainless steel. Before the LDI MS and MS imaging experiments, steel targets were cleaned through soaking in boiling solvents: toluene (3 × 100 mL, each plate for 30 s), chloroform (3 × 100 mL, each plate for 30 s), acetonitrile (3 × 100 mL, each plate for 30 s), and deionized water (3 × 100 mL, each plate for 30 s). Every plate was dried in high vacuum (ca. 0.01 mbar, 24 h). Optical photographs were made with the use of an Olympus SZ10 microscope equipped with an 8 MPix Olympus digital camera and also a Canon 6D camera with a macro-type 90 mm focal length lens.

### PFL 2D GS Laser Ablation Synthesis in Solution (LASiS) of Silver-109 Nanoparticles

**Figure 1:**
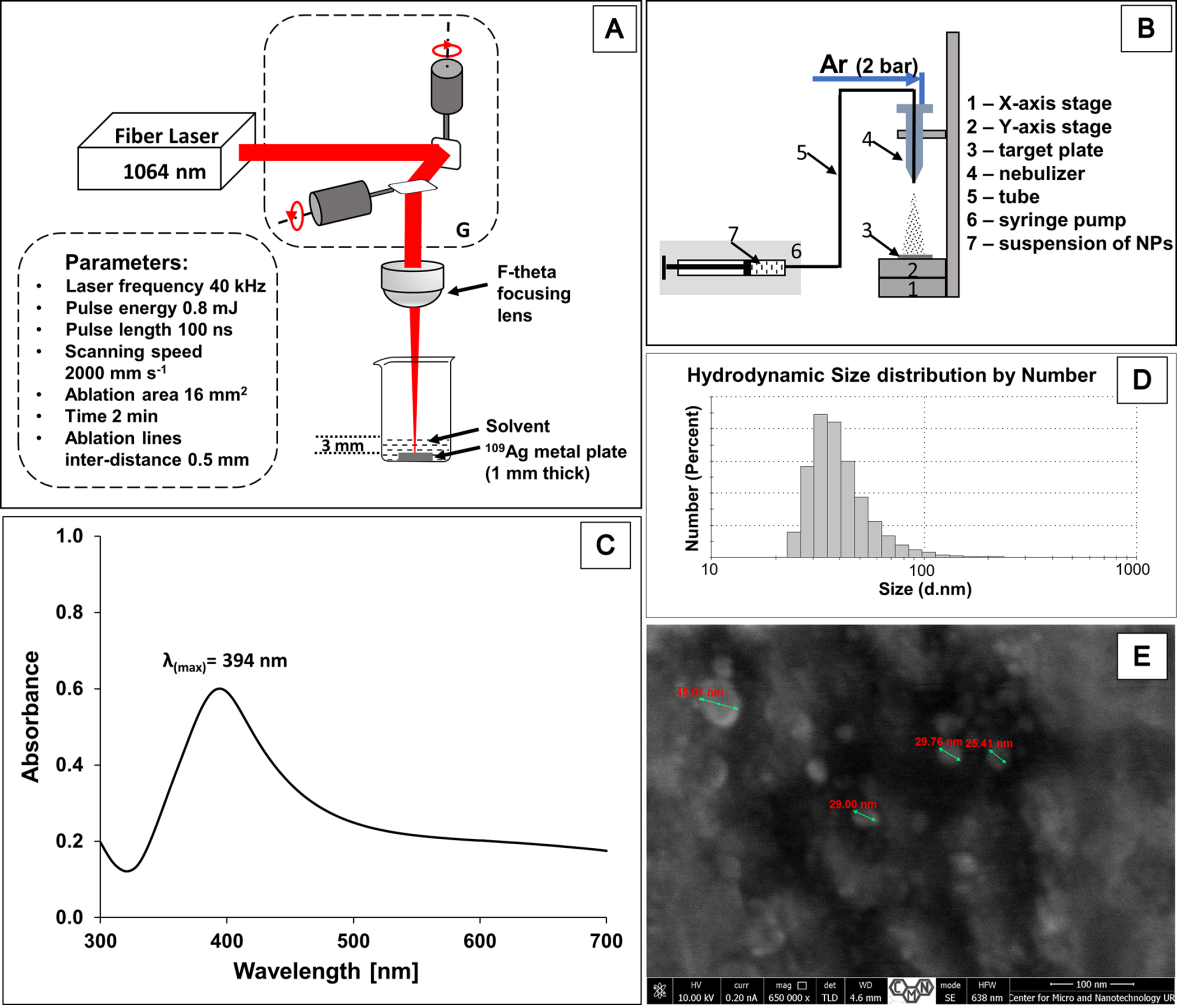
(A) Laser ablation setup for the preparation of ^109^AgNPs; G – 2D galvanometer laser scanner. Right panel (B) presents setup for nebulization of nanoparticles. Panel (C) presents UV–vis spectrum of ^109^AgNP suspension in acetonitrile. Panel (D) shows results of DLS measurement of ^109^Ag nanoparticles’ hydrodynamic size distribution by number. (E) High-resolution SEM image of target modified with ^109^AgNPs generated by PFL 2D GS (three sizes in the center are 29.00, 29.76, and 25.41 nm).

The experimental arrangement for the ^109^AgNP preparation by laser ablation is shown in Figure 1A. The silver-109 foil (∼1 mm thick) was placed at the bottom of a glass vessel containing solvent (acetonitrile or isopropanol). The ^109^Ag foil was covered by an approximately 3 mm thick layer of solvent (total solvent volume was 3 mL). The laser ablation was carried out with a 1064 nm pulsed fiber laser (Raycus RFL-P20QE/A3). A suspension was obtained after 2 min of irradiation with a pulse energy of 0.8 mJ (100 ns pulse length) at a 40 kHz repetition rate. Laser ablation was accomplished at a scanning speed of 2000 mm/s; the ablation area was 4 × 4 mm. The suspension was immediately transferred into a syringe and used in the nebulization step.

### Nebulization of ^109^AgNP Suspension

The experimental setup for the nebulization of ^109^AgNP suspension is shown in Figure 1B. The entire nanoparticle nebulization process was controlled by a computer. The H17 steel plate (laser mass spectrometry target plate) was placed on the table of a translation system consisting of a motorized XY table (EzM-42XL-A powered by closed-loop Ezi-SERVO motors). A glass syringe (1 mL) was filled with a previously prepared suspension of silver-109 nanoparticles and placed in a syringe pump (pumping speed 250 μL/min). The custom-made software directed the 2D system table with 10 mm/s speed using a sequence of movement designed to uniformly cover a target plate. The nebulizer was obtained from a Bruker Amazon ETD ESI ion source. Argon at a pressure of 2 bar was used as the nebulizing gas. Generally, all studied objects—for MS and MSI—were placed on the target plate before nebulization.

### ^109^AgNP Characterization

The ^109^AgNP suspension was characterized by UV–vis spectroscopy (Jasco V-670 spectrophotometer). The spectrum was registered in quartz cuvettes within a 200–800 nm spectral range. The blank sample contained acetonitrile. The suspension of ^109^AgNPs was also characterized by dynamic light scattering (DLS) using a Zetasizer-Nano ZS from Malvern Instruments. DLS measurements were performed by backscattering at a fixed detector angle of 173°. Isopropanol was used as a dispersant.

### LDI MS Experiments

LDI-ToF mass spectrometry experiments were performed using a Bruker Autoflex Speed ToF mass spectrometer equipped with a SmartBeam II laser (355 nm). The laser pulse energy was approximately 90–140 μJ, and the laser repetition rate was 1 kHz. Compounds were measured within 80–1500 or 80–2000 *m*/*z* windows, and ion deflection was turned on for ions lighter than *m*/*z* 79. The first accelerating voltage was held at 19 kV, and the second ion source voltage was held at 16.7 kV. The reflector voltages used were 21 and 9.55 kV. The spectra for histidine, thymidine, and ribose were acquired by integrating approximately 4000 shots, for which the PPG 5000 shots package was used. Spectra were internally calibrated and analyzed with FlexAnalysis (version 3.3). Mass calibration (typically enhanced cubic calibration based on 5–10 points) was performed using internal standards (silver ions and clusters from ^109^Ag^+^ to ^109^Ag_10_^+^).

### LDI MS Imaging Experiments

Measurements were performed using a Bruker Autoflex Speed time-of-flight mass spectrometer in reflectron mode. The apparatus was equipped with a SmartBeam II 1000 Hz, 355 nm laser. The laser impulse energy was approximately 90–140 μJ, the laser repetition rate was 1 kHz, and deflection was used for *m*/*z* lower than 80 Da. The *m*/*z* range was 80–1500 for the fingerprint experiment (40 × 40 μm spatial resolution). The first accelerating voltage was held at 19 kV, and the second ion source voltage was held at 16.7 kV. Reflector voltages used were 21 kV (the first) and 9.55 kV (the second). The experiments were made with 1000 laser shots per individual spot with random walk applied (FlexImaging 4.0). A random spot measurement pattern was used for all MSI experiments. All spectra were calibrated with the use of silver ions (^109^Ag^+^ to ^109^Ag_10_^+^). All of the generated ion images were within the ±0.05% *m*/*z* range. TIC normalization was used for all results shown.

### LDI Sample Preparation

Stock solution (0.1 mg/mL) of each analyte was prepared by dissolving it in water (histidine, ribose, thymidine). In order to prepare analyte solutions of lower concentrations, the stock solution was diluted with ultrapure water. A solution of poly(propylene glycol) in isopropanol of a 10 μg/mL concentration was prepared. A 0.5 μL volume of each of the final solutions was applied to the steel target and air-dried followed by nebulization with the ^109^AgNP suspension.

### MALDI Sample Preparation

MALDI experiments were performed using a DHB matrix solution (saturated matrix in acetonitrile with 0.5% of trifluoroacetic acid) by the drying droplet method (1:1 v/v matrix:sample solution). A volume of 1 μL of sample mixed with matrix solution was placed directly on steel plate and air-dried, and the target was inserted into an MS apparatus for measurement. Calibration was performed on matrix signals.

### Imaging Sample

Preparation of the ungroomed fingerprint for mass spectrometry imaging was obtained by touching the clean steel target for approximately 1 s. Then, the object was covered with a layer of nanoparticles by nebulization, as described in paragraph 2.3.

### High-Resolution Scanning Electron Microscopy (HR SEM)

A target modified with ^109^AgNPs generated by a PFL 2D GS method was inserted into the Helios Nanolab 650 electron microscope. The voltage was set at 10 and 30 kV, and the current was set as 0.2 nA. Images were made in nonimmersive mode.

## Results and Discussion

### PFL 2D GS LASiS of ^109^AgNPs

The laser mass spectrometry usually is realized via MALDI methodology. It employs organic low-molecular-weight matrices, such as α-cyano-4-hydroxycinnamic acid (CHCA) and 2,5-dihydroxybenzoic acid (DHB). This methodology is preferred only for ionic substances, such as peptides and proteins of molecular weights higher than 1000 Da due to (i) numerous matrix signals in the region of *m*/*z* < 1000, (ii) unreliable calibration, (iii) low mass accuracy, (iv) low ionization potential for neutral organic compounds, and (v) the sweet-spot effect. Moreover, due to the acidity of the standard matrix solutions, the analysis of various substances may be problematic.^31^

Most of above-mentioned MALDI problems may be solved by using metal nanoparticles, for example, silver ones, as desorption/ionization agents.^16,21,32^ Silver nanoparticles were produced by different means including chemical, physical, and biological methods. Chemical synthesis is most commonly used to obtain AgNPs. The reaction involves the reduction of Ag^+^ to elemental silver by electron transfer under various conditions. The chemical reduction method requires two substances: a metal salt precursor and a reducing agent. However, almost all procedures contain also a stabilizer. Among the many silver precursors, we can distinguish silver nitrate, silver ammonia complexes, and silver sulfate. In turn, the role of the reducing agent is often assumed by sodium borohydride or sodium citrate. Both the type of precursor and reductant used can influence the properties of the AgNPs obtained.^21^ Preparation of silver nanoparticles by a chemical reduction method for laser mass spectrometry was presented by Hua and co-workers. Authors used silver nitrate as the metal precursor and sodium cyanoborohydride as the reducing agent. AgNPs were used as matrices for studying the MALDI MS of peptides such as bradykinin and angiotensin I. The obtained results present only proton adducts of the mentioned compounds along with a relatively high noise level.^33^ In contrast, Sherrod et al. used commercially available 20 and 60 nm silver nanoparticles to ionize peptides and observed no signals on the MS spectrum.^34^ Ding and co-workers used a chemical reduction method to obtain silver nanoparticles of different sizes. Silver nitrate or silver perchlorate was used as the metal precursor, while sodium citrate and/or sodium borohydride was used as the reducing agent. The AgNPs obtained were used as a matrix for amyloid-beta peptide MALDI-ToF-MS measurements. However, the processes of purifying the suspension from reaction byproducts or unreacted substrates make this method of producing silver nanoparticles very time-consuming and complicated.^35^

The above-mentioned problems were partly solved by Yonezawa and co-workers, who were the first to demonstrate a method for producing nanoparticles by laser ablation in an aqueous medium and their applicability as a matrix for laser MS.^24^ Compared to chemical synthesis, laser ablation synthesis in solution has some very unique properties including: (i) cost-effectiveness, (ii) simplicity, (iii) time-efficiency, (iv) spectrum simplicity and low chemical background, (v) the ability to prepare NPs from a variety of metals or alloys, and (vi) in situ dispersion of the nanoparticles in a variety of liquids. In general, LASiS produces suspensions of a relatively high chemical purity as compared to chemical methods.

In this work, LASiS has been used to obtain chemically pure monoisotopic silver-109 nanoparticles. Natural silver contains two isotopes: ^107^Ag (ca., 51.8%) and ^109^Ag (ca., 48.2%). It is logical to state that the use of monoisotopic silver-based MS methods provide analyte peaks that are roughly 2-fold higher in intensity compared to normal silver. Signals based on a single silver-109 isotope have also a higher signal-to-noise (S/N) ratio. What is more, internal calibration with the use of silver-109 signals is greatly improved due to many times higher intensity and S/N of complex Ag_*x*_^+^ (*x* = 2–30) ions compared to normal silver.

Nanoparticles were generated by a pulsed 1064 nm fiber laser with galvoscanner head scanning of an ablated surface. The experimental setup for the ^109^AgNP preparation is shown in Figure 1A. For fast synthesis of nanoparticles, a high-frequency (60–80 kHz), high-pulse-energy (up to 1 mJ/pulse) laser was used. However, in order to avoid unwanted thermal effects such as melting, solvent boiling, and oxidation of solvent and also of nanoparticles, a two-dimensional (2D) galvoscanner (GS) was used. A galvoscanner head with an f-theta lens attached to a fiber laser allowed for precise and very fast shifting of a focused laser beam on the surface of an ablated metal foil.

Prepared nanoparticles were first studied by UV–vis spectroscopy. Particular wavelengths of light can induce the metallic electrons to oscillate, which causes an effect known as surface plasmon resonance (SPR). It is associated with a specific size and shape of the silver nanoparticles as well as chemical surroundings. Therefore, the UV–vis spectroscopy method can be of some aid in determining the size and shape of nanoparticles. The literature describes that as the diameter of AgNPs increases, the absorbance band shifts toward longer wavelengths and also broadens.^36,37^ The UV–vis spectrum obtained for PFL 2D GS LASiS of ^109^AgNPs is shown in Figure 1C. The ^109^AgNP postreaction suspension UV–vis spectrum recorded after 3 min of synthesis contains a local maximum at 394 nm, which suggests that the size of most of nanoparticles is approximately 10 nm. However, one can observe an asymmetric broadening of the SPR toward longer wavelengths, which is characteristic for a fraction of spheroidal particles. The occurrence of spheroids may indicate particle aggregation processes taking place in the suspension.^29^ Similar results were reported by several authors.^21,29,36^

Figure 1D presents the results of dynamic light scattering (DLS) measurement of ^109^AgNP size distribution by number after a few minutes after preparation of a suspension. The DLS chart of the size distribution by number indicates the highest content of nanoparticles being around 30 nm in diameter, with a distribution ranging from 20 to 100 nm. A high-resolution scanning electron microscope image of a modified target (Figure 1E) also confirms that individual nanoparticles are in roughly round/spherical shape and are of 25–35 nm size. A number of HRTEM and DLS results on size measurements of silver nanoparticles obtained with LASiS in different organic solvents indicate that NPs with a size of ∼10 nm are the most common group.^21,29^ DLS results suggest bigger nanoparticles as judged from a UV–vis spectrum. Most probably, this is due the fact that the nanoparticle suspension used for the DLS measurement was prepared in a different solvent than the one optimized for LASiS. Many studies show the effect of solvent on the size of nanoparticles obtained.^29^

Application of nanoparticles in laser mass spectrometry requires a suitable method of application on the surface containing a studied object, which may be, for example, a sample spot or tissue slice. One approach of using AgNPs as a matrix is dry metal sputtering, which allows for preparation of a homogeneous layer with minimal or no lateral migration of the analyte on the laser beam size scale.^38^ Silver deposition by sputtering has been applied to various types of samples, including fingerprints,^39^ but also to a human carotid^40^ or samples of colorectal cancer metastases to the liver.^41^ However, the prevalence of this method is low, probably due to the need for a specialized sputtering system. Moreover, sputtering requires that a sample is treated in very high vacuum, which can be problematic for some of tissues due to warping and cracking. Yang, Fournelle, and Chaurand presented silver-assisted laser desorption ionization (AgLDI) MSI, where a silver salt solution was sprayed to obtain a homogeneous layer on thin tissue sections. However, the method did not achieve the same high spatial resolution as Dufresne et al. or other researchers.^38,42^

**Figure 2:**
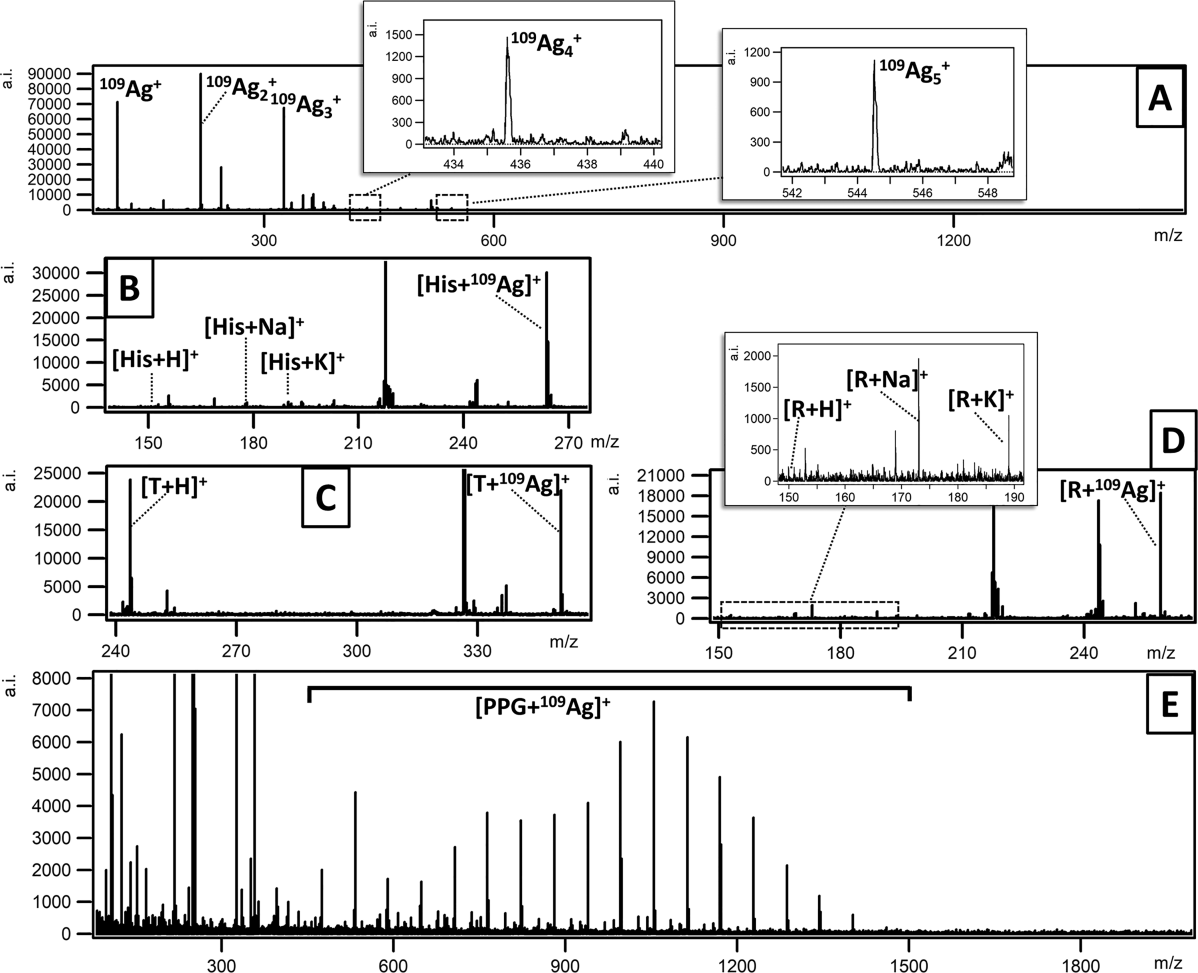
LDI MS positive reflectron mode spectrum of target plate covered with ^109^AgNPs generated by PFL 2D GS (A). Others panels present LDI MS spectra fragments for histidine (B), thymidine (C), and ribose (D) of 0.1 mg/mL concentrations deposited on the target plate and covered with silver-109 nanoparticles obtained with PFL 2D GS LASiS. The last panel (E) presents an LDI MS spectrum of poly(propylene glycol) of a 10.0 μg/mL concentration spot at the same target as the above-mentioned compounds.

For our LDI MS measurements, 0.5 μL of each of solution of histidine, ribose, thymidine, and PPG polymer was applied to a stainless-steel plate and air-dried. The plate with all test objects was placed on the table of the translation system as shown in Figure 2B. Aliquots of colloidal silver-109 (1 mL) were sprayed three times onto the sample. Each portion was injected into the nebulizer at a constant rate of 250 μL/min. The entire nanoparticle nebulization process was controlled by a computer using a sequence of movements aimed at evenly covering the target plate.

The LDI MS spectrum of ^109^AgNPs produced by PFL 2D GS LASiS and deposited on the surface of stainless steel of target plate by nebulization is shown in Figure 2A. The mass spectrum made in the 80–1500 *m*/*z* range contains virtually only silver-109 ion peaks of ^109^Ag^+^ to ^109^Ag_10_^+^ composition. Various low-molecular-weight compounds such as ionic amino acid histidine and nonionic ribose and thymidine were tested to verify the potential of ionization of organic compounds with the silver-109 nanoparticles obtained with PFL 2D GS LASiS.

The first analyzed compound histidine (Figure 2B) was found mainly as a ^109^Ag adduct but sodium, potassium, and protonated adducts signals were also found. Histidine was recently analyzed on diamond nanowires acting as an LDI active surface, presenting a spectrum with a number of unknown signals in the *m*/*z* 50–250 region.^43^ Our previous attempt to analyze histidine was made using a ^109^AgNPET target. The S/N ratio of the histidine–^109^Ag adduct for ^109^AgNPET was 214,^44^ while for PFL 2D GS LASiS, ^109^AgNPs is higher at 280. What is more, the target covered by PFL 2D GS LASiS ^109^AgNPs that is ready for mass spectrometry can be made within 10 min of time, which is in contrast to our previous method ^109^AgNPET, where target preparation took 48 h.

Thymidine was also tested as a very good example of a biologically important, medium-polarity nonionic compound. The LDI MS spectrum of thymidine (T; 0.1 mg/mL) with PFL 2D GS LASiS ^109^AgNPs in reflectron positive mode is shown in Figure 2C. The thymidine MS spectrum obtained with the use of PFL 2D GS LASiS ^109^AgNPs shows two highest peaks, assigned to a thymidine–silver adduct with an S/N ratio of 138 and protonated with an S/N ratio of 175. Measuring nucleosides using modified graphene as a matrix was shown by Wang et al.; however, the results presented a low S/N ratio.^45^

Another compound tested was ribose, a nonionic medium-polarity saccharide. The LDI MS spectrum within *m*/*z* 80–1500 in reflectron positive mode of ribose on a target covered by PFL 2D GS LASiS ^109^AgNPs is shown in Figure 2D. The highest peak visible in the spectrum belongs to the ribose–^109^Ag^+^ adduct. Protonated, potassium, and sodium adducts were also visible, and the S/N ratios for protonated, sodium, potassium, and ribose–^109^Ag adducts were 3, 23, 12, and 150, respectively. Bibi and Ju utilized quantum dots (QDs) with some modifications as a matrix for LDI-TOF MS to small monosaccharides including ribose.^46^ In contrast, Zhang and colleagues showed that the use of traditional MALDI matrices such as DHB or CHCA is not suitable for the analysis of small oligosaccharides such as ribose. The matrix-derived peaks were dominant on the spectrum and were the cause of ribose signal suppression.^47^

**Figure 3:**
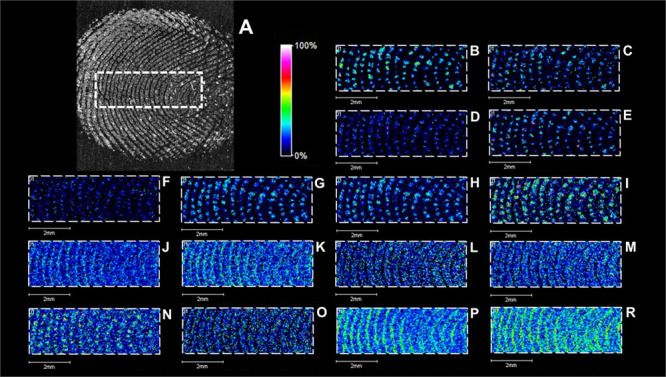
Results of LDI MS imaging of a fingerprint with PFL 2D GS LASiS ^109^AgNPs. Optical microscope images of a fingerprint (A). Images (B–R) (TIC-normalized) represent spatial distribution of ions of *m*/*z* 96.922 (B), 98.996 (C), 106.050 (D), 148.061 (E), 178.0592 (F), 183.078 (G), 183.175 (H), 185.069 (I), 240.947 (J), 241.942 (K), 283.264 (L), 311.295 (M), 333.119 (N), 334.283 (O), 363.324 (P), and 391.355 (R). Spatial resolution 40 × 40 μm.

Poly(propylene glycol) is a compound belonging to the polymer group, consisting of propylene oxide-mers. The repeating monomer unit mass of approximately 57.9 is of the CH_2_CH(CH_3_)O chemical formula. The LDI mass spectrum of PPG is shown in Figure 3E. As can be seen, the spectrum contains a typical polymer structure with a dominating mass of approximately *m*/*z* 1000, which positively corroborates with the polymer used. For example, the highest polymer signal at *m*/*z* 1055 revealed is the ^109^Ag^+^ ion adduct of a PPG with 16-mer units. Comparison of polypropylene glycol spectra between MALDI and SALDI were made by Okuno et al. The authors identified a problem with the reproducibility of MALDI mass spectra for PPG, which showed a strong dependence on the analyte/matrix ratio and on the type of solvent and/or chemical matrix.^48^

Comparison of the new method with previously published ones is shown in Table 1. The upper part of table presents comparison of *m*/*z* matching errors (calculated and experimental) of a few test low-molecular-weight compounds shown as ppm values. Very big, unacceptable *m*/*z* errors of MALDI are surely an effect of calibration performance. The silver-method-based spectrum was calibrated with the use of nine signals, while the MALDI one was calibrated with only two matrix signals. It should be noted that the ribose signal is marked as “not found” as a big *m*/*z* difference and low intensity did not allow it to be assigned. The lower part of Table 1 contains comparison of previously published method ^109^AgNPET that is based on the chemical synthesis of nanoparticles with the one based on PFL 2D GS LASiS. It is clearly seen that PFL 2D GS LASiS ^109^AgNPs produce much higher signals under laser irradiation, which in turn allows better calibration, especially in the higher *m*/*z* region.

**Table 1:** Comparison of Mass Spectrometry Data of PFL GS LASiS Nanoparticles with Chemically Synthesized Ones (AgNPET) and Also with MALDI

calculated–experimental *m*/*z* errors in ppms		
	Δ *m*/*z* [ppm]	
compound	MALDI	PFL 2D GS LASiS
histidine	184	8
thymidyne	314	12
ribose	not found	7
3-methylhippuric acid	1207	21
alternariol	77	8
comparison of signal intensity		
	^109^AgNPET [16]	PFL 2D GS LASiS ^109^AgNPs
^109^Ag^+^	14 453	59 969
^109^Ag_2_^+^	21 351	89 591
^109^Ag_3_^+^	19 818	77 933
^109^Ag_4_^+^	386	1810
^109^Ag_5_^+^	785	10 579

### MS Imaging with PFL 2D GS LASiS ^109^AgNPs

The fingerprint was chosen as a test subject to determine the applicability of PFL 2D GS LASiS ^109^AgNPs for imaging exogenous and endogenous compounds on a human finger. Francese and co-workers^49^ were the first to demonstrate the applicability of MALDI MSI for fingerprint trace analysis. The fingerprint is one of the most important means of biometric identification, as it is a source of both physical and chemical information. The physical information provided by a fingerprint is the geometry, distribution, and size of sweat pores and also local shapes such as terminations, bifurcations, islands, spurs, etc. Chemical information found on fingerprints includes exogenous and endogenous substances, including drugs, explosives, toxins, poisons, cosmetics, toiletries, etc. Endogenous compounds found on the skin include lipids, peptides, amino acids, proteins, urea, simple inorganic compounds, as well as organic salts.

The LDI MS imaging experiment involved a fingerprint left on the stainless-steel surface. Preparation of the fingerprint is extremely simple, and only requires a finger to touch the target surface. A suspension of monoisotopic silver-109 nanoparticles was sprayed onto the obtained fingerprint. Table 2 contains names and ion data for some of the compounds found in the imaging experiment. Ion images for some of the ions from Table 2 are shown in Figure 3 and the Supporting Information (S1).

**Table 2:** Compounds and Their Ions Found in MSI Experiment

	compound^a^	ion formula	*m*/*z*_calc._^b^	
1.	KCl	[KCl + Na]^+^	96.9223	Figure 3B
2.	urea	[CH_4_N_2_O + K]^+^	98.9961	Figure 3C
3.	1-hexanoic acid	[C_6_H_12_O_2_–H_2_O + H]^+^	99.0810	S1.A
4.	serine	[C_3_H_7_NO_3_ + H]^+^	106.0504	Figure 3D
5.	1-hexanoic acid	[C_6_H_12_O_2_ + H]^+^	117.0916	S1.B
6.	l-cysteine	[C_3_H_7_NO_2_S + H]^+^	122.0276	S1.C
7.	l-glutamic acid	[C_5_H_9_NO_4_ + H]^+^	148.0610	Figure 3E
8.	2-aminoadipic acid	[C_6_H_10_O_4_ + Na]^+^	169.0477	S1.D
9.	asparagine	[C_4_H_8_N_2_O_3_ + K]^+^	171.0172	S1.E
10.	histidine	[C_6_H_9_N_3_O_2_ + Na]^+^	178.0592	Figure 3F
11.	octanoic acid	[C_8_H_16_O_2_ + K]^+^	183.0787	Figure 3G
12.	dodecanoic acid	[C_12_H_24_O_2_–H_2_O + H]^+^	183.1749	Figure 3H
13.	2-aminoadipic acid	[C_6_H_10_O_4_ + K]^+^	185.0216	S1.F
14.	l-lysine	[C_6_H_14_N_2_O_2_ + K]^+^	185.0692	Figure 3I
15.	pyruvic acid	[C_3_H_4_O_3_ + ^109^Ag]^+^	196.9208	S1.G
16.	glyceric acid	[C_3_H_6_O_4_ + ^109^Ag]^+^	214.9313	S1.H
17.	pentyl 2-hydroxybenzoate	[C_12_H_16_O_2_ + Na]^+^	215.1048	S1.I
18.	glutaric acid	[C_5_H_8_O_4_ + ^109^Ag]^+^	240.9470	Figure 3J
19.	asparagine	[C_4_H_8_N_2_O_3_ + ^109^Ag]^+^	240.9582	S1.J
20	aspartic acid	[C_4_H_7_NO_4_ + ^109^Ag]^+^	241.9422	Figure 3K
21.	phenylacetic acid	[C_8_H_8_O_2_ + ^109^Ag]^+^	244.9572	S1.K
22.	3-oxoglutaric acid	[C_5_H_6_O_5_ + ^109^Ag]^+^	254.9262	S1.L
23.	mevalonic acid	[C_6_H_12_O_4_ + ^109^Ag]^+^	256.9783	S1.M
24.	ribose	[C_5_H_10_O_5_ + ^109^Ag]^+^	258.9575	S1.N
25.	9-octadecenoic acid	[C_18_H_34_O_2_ + H]^+^	283.2637	Figure 3L
26.	*cis*-13-eicosenoic acid	[C_20_H_38_O_2_ + H]^+^	311.2950	Figure 3M
27.	pentadecenoic acid	[C_15_H_28_O + ^109^Ag]^+^	333.1187	Figure 3N
28.	methyl linoleate	[C_19_H_34_O_2_ + K]^+^	333.2196	S1.O
29.	*cis*-13-eicosenoic acid	[C_20_H_38_O_2_ + Na]^+^	333.2770	S1.P
30.	*N*-dodecyl-4-methyl-1-piperazine carboxamide	[C_18_H_37_N_3_O + Na]^+^	334.2834	Figure 3O
31.	tricosanoic acid	[C_23_H_46_O_2_–H_2_O + H]^+^	337.3470	S1.R
32.	butyl octadecanoate	[C_22_H_44_O_2_ + Na]^+^	363.3239	Figure 3P
33.	tetracosanoic acid	[C_24_H_48_O_2_ + Na]^+^	391.3552	Figure 3R

a Putative identification.

b *m*/*z*_calc_. – calculated monoisotopic *m*/*z* value.

The compounds that were identified in the studied fingerprint belong to different groups such as inorganic salts (e.g., NaCl, KCl), simple organic compounds (e.g., urea, amino acids, short carboxylic acids), fatty acids, lipids, and others. Most of these compounds are considered endogenous, secreted through the skin or sweat pores.

Ion images for ions presented in Table 2 are shown in Figure 3. In the fingerprint, six amino acids such as serine, cysteine, glutamic acid, asparagine, histidine, and lysine were detected. Figure 3 contains four images showing the spatial distribution of amino acids with the following *m*/*z* values of 106.050 (D), 148.061 (E), 178. 0592 (F), and 185.0692 (I), which were assigned to protonated serine, protonated glutamic acid, a histidine–sodium adduct, and a lysine–potassium adduct, respectively. As judged from the ion image, these amino acid ions are found in close proximity to sweat pores, which produce round structures rich in the mentioned ions. Numerous studies show that amino acids/proteins are one of the most numerous groups of compounds present in a fingerprint.^50^Figure 3 also contains images showing ions with relatively low *m*/*z*, such as 96.922 (B) and 98.996 (C) assigned to adduct [KClNa]^+^ and a urea–potassium adduct. These ions correspond to compounds secreted directly by sweat pores (KCl/NaCl and urea) that can be used to localize sweat pores as well as dorsal patterns.

Another group of compounds that were detected in the fingerprint were free fatty acids such as octanoic, dodecanoic, octadecenoic acid, eicosenoic, pentadecenoic, and tetracosanoic acids. Figure 3 presents ion images showing the spatial distribution of mentioned fatty acids with the following *m*/*z* values of 183.0787 (G), 183.1749 (H), 283.2637 (L), 311.2950 (M), 333.1187 (N), and 391.3552 (R), which are attributed to an octanoic acid–potassium adduct, protonated dodecanoic acid with water molecule loss, protonated 9-octadecenoic acid and protonated *cis*-13-eicosanoic acid, a pentadecenoic acid–silver-109 adduct, and a tetracosanoic acid–sodium adduct, respectively. The mentioned ions are presenting very clearly representations of fingerprint ridges. Free fatty acids were previously identified in a fingerprint using different analytical techniques such as MALDI or SALDI MS.^50^

## Conclusions

A novel method for synthesis and application of monoisotopic silver-109 nanoparticles onto a studied surface for LDI MS and MSI is presented. The methodology was proven to be very useful for analysis and MS imaging of low-molecular-weight (LMW) compounds and polymers as well as for mass spectrometry imaging. LASiS with the use of 1064 nm pulsed nanosecond fiber laser on galvomotors allowed highly efficient synthesis of nanoparticles of extremely high chemical purity. LASiS of ^109^AgNPs coupled with nebulization was used for surface-transfer mass spectrometry imaging of a fingerprint, allowing investigation of ridge patterns and sweat pores as well as determination of spatial distribution of compounds.

## Supporting Information Available

Ion images of fingerprint (PDF)
