# Supplementary material for: Laser Ablation Synthesis in Solution and Nebulization of Silver-109 Nanoparticles for Mass Spectrometry and Mass Spectrometry Imaging
Source: ACS Meas Sci Au. 2021 Aug 25;2(1):14–22. doi: 10.1021/acsmeasuresciau.1c00020 (PMC9885948; doi:10.1021/acsmeasuresciau.1c00020)
Supplement: Supplementary file 1 — tg1c00020_si_001.pdf [file tg1c00020_si_001.pdf]

Supporting Information

Laser ablation synthesis in solution and nebulization of silver-109 nanoparticles for mass spectrometry and mass spectrometry imaging.

Aneta Płaza[a], Artur Kołodziej[a], Joanna Nizioł[b], and Tomasz Ruman[b]*

[a]*Doctoral School of Engineering and Technical Sciences at the Rzeszów University of Technology, 8 Powstańców Warszawy Ave., Rzeszów, 35-959, Poland*

[b]*Rzeszów University of Technology, Faculty of Chemistry, Bioorganic Chemistry Laboratory, 6 Powstańców Warszawy Ave., 35-959 Rzeszów, Poland*

Content: ion images of fingerprint

S1. Ion images of fingerprint obtained with the use of PFL 2D GS LASIS ^{109}AgNPs and nebulization. Images (TIC normalized) represent spatial distribution of ions of various *m/z* (visible in images). Spatial resolution 40 x 40 µm.

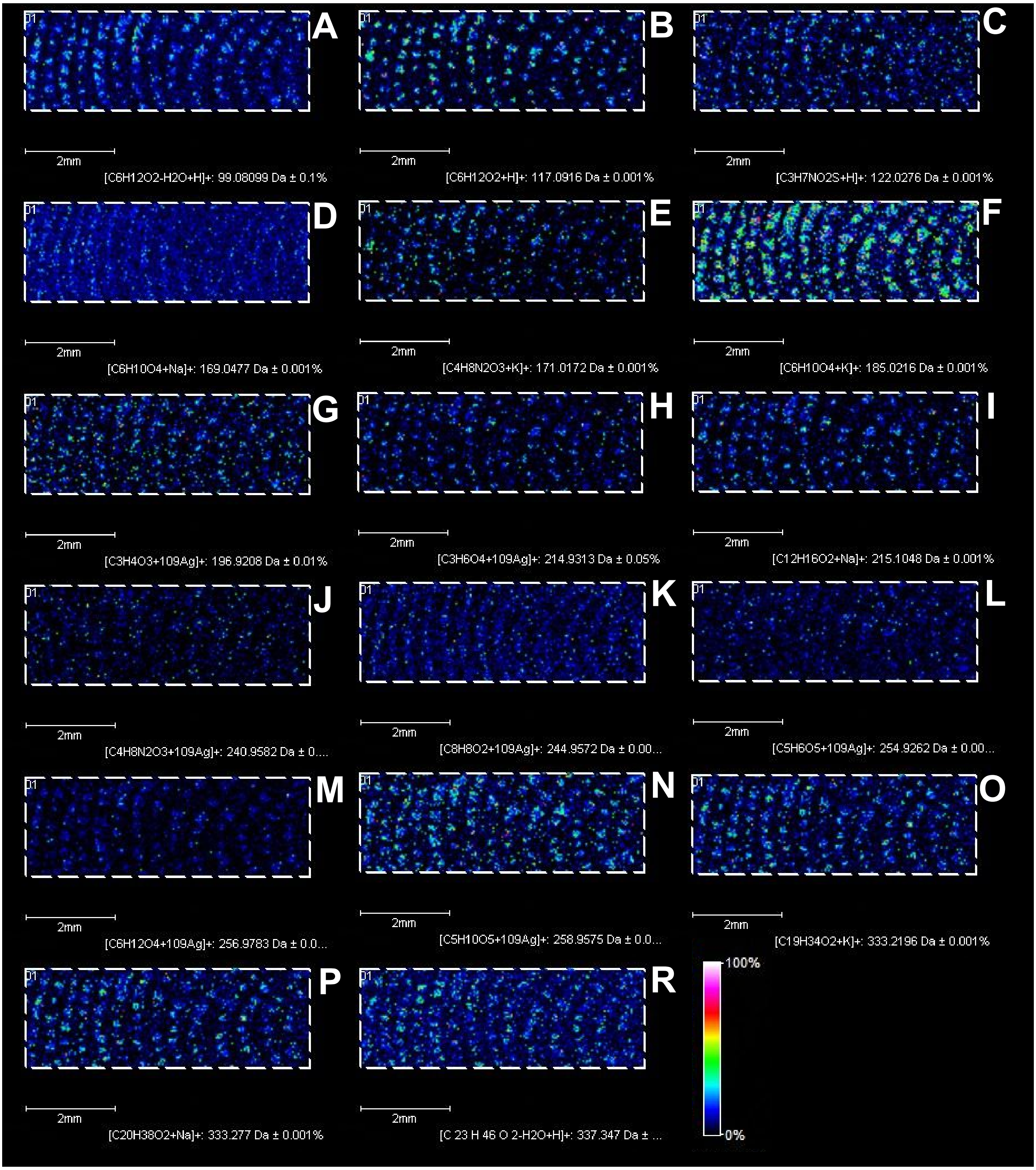

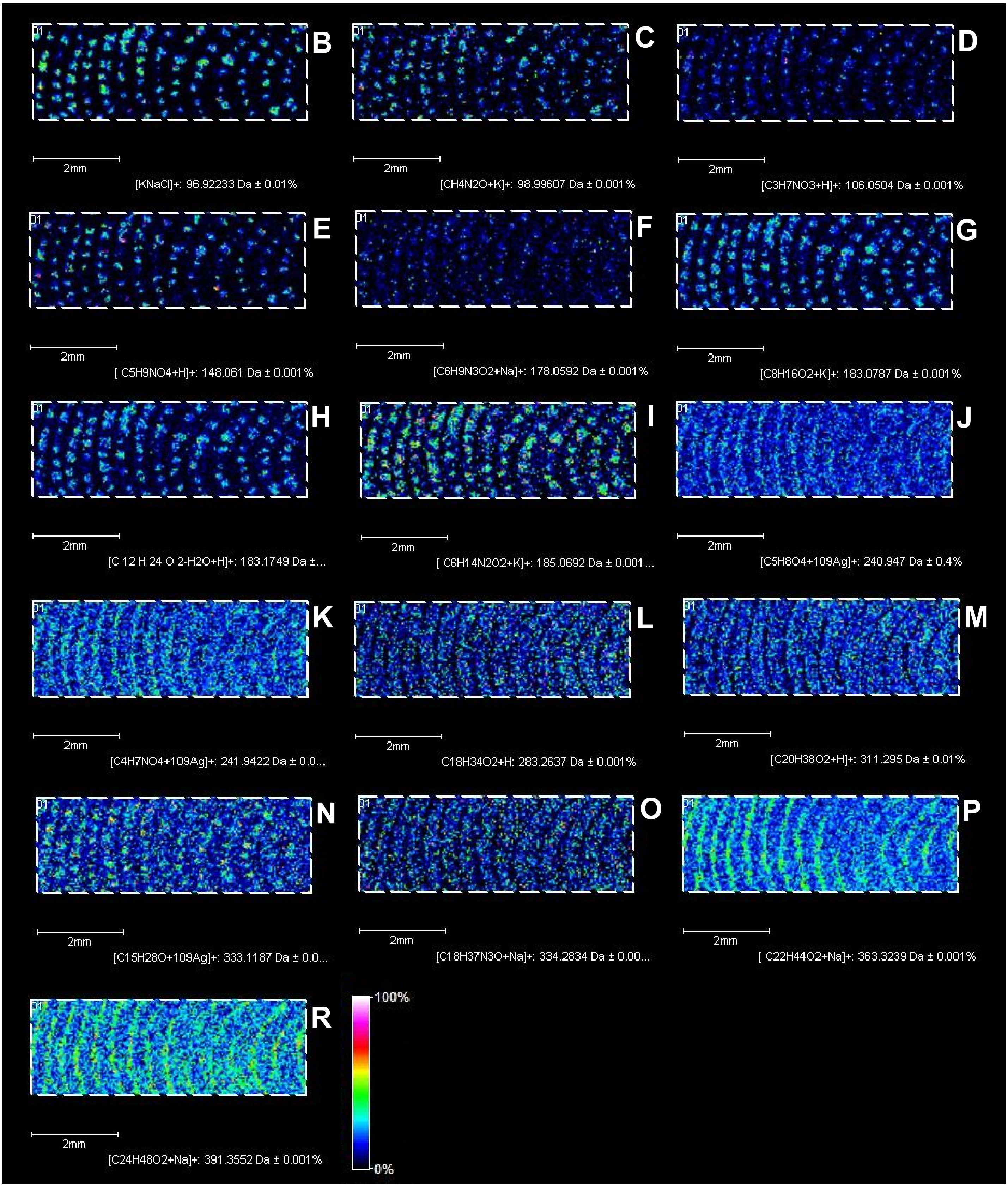
B
2mm
[KNaCl]+: 96.92233 Da ± 0.01%
C
2mm
[CH4N2O+K]+: 98.99607 Da ± 0.001%
D
2mm
[C3H7NO3+H]+: 106.0504 Da ± 0.001%
E
2mm
[C5H9NO4+H]+: 148.061 Da ± 0.001%
F
2mm
[C6H9N3O2+Na]+: 178.0592 Da ± 0.001%
G
2mm
[C8H16O2+K]+: 183.0787 Da ± 0.001%
H
2mm
[C 12 H 24 O 2-H2O+H]+: 183.1749 Da ±...
I
2mm
[C6H14N2O2+K]+: 185.0692 Da ± 0.001...
J
2mm
[C5H8O4+109Ag]+: 240.947 Da ± 0.4%
K
2mm
[C4H7NO4+109Ag]+: 241.9422 Da ± 0.0...
L
2mm
C18H34O2+H: 283.2637 Da ± 0.001%
M
2mm
[C20H38O2+H]+: 311.295 Da ± 0.01%
N
2mm
[C15H28O+109Ag]+: 333.1187 Da ± 0.0...
O
2mm
[C18H37N3O+Na]+: 334.2834 Da ± 0.00...
P
2mm
[C22H44O2+Na]+: 363.3239 Da ± 0.001%
R
2mm
[C24H48O2+Na]+: 391.3552 Da ± 0.001%
100%
0%

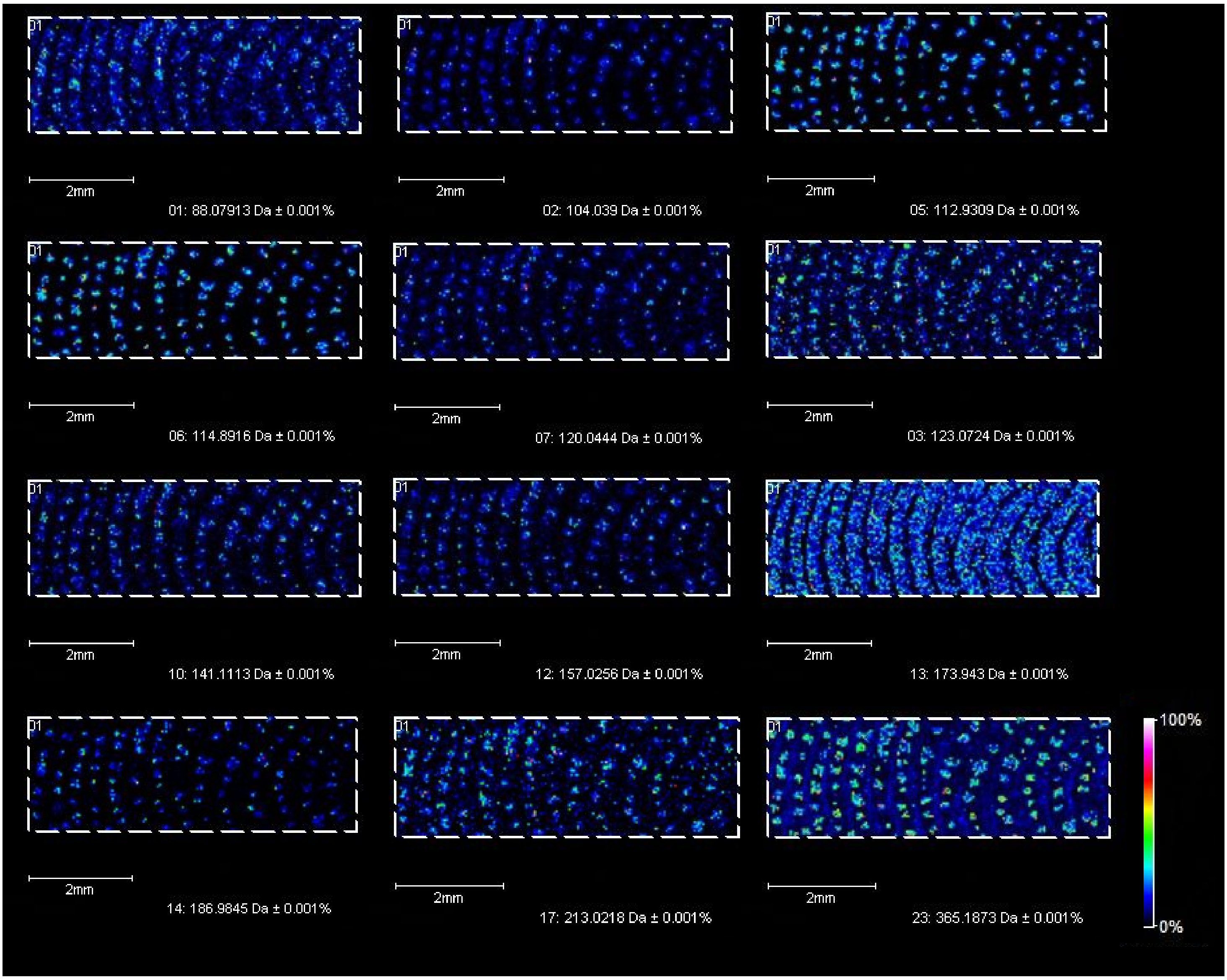
D1
2mm
01: 88.07913 Da ± 0.001%
02: 104.039 Da ± 0.001%
05: 112.9309 Da ± 0.001%
06: 114.8916 Da ± 0.001%
07: 120.0444 Da ± 0.001%
03: 123.0724 Da ± 0.001%
10: 141.1113 Da ± 0.001%
12: 157.0256 Da ± 0.001%
13: 173.943 Da ± 0.001%
14: 186.9845 Da ± 0.001%
17: 213.0218 Da ± 0.001%
23: 365.1873 Da ± 0.001%
100%
0%
